# Research on Cross-Contrast Neural Network Based Intelligent Painting: Taking Oil Painting Language Classification as an Example

**DOI:** 10.1155/2022/7827587

**Published:** 2022-06-06

**Authors:** Xi Zeng

**Affiliations:** Oil Painting Department, School of Drawing and Art, Guangzhou Academy of Fine Art, Guangzhou 123456, Guangdong, China

## Abstract

With the continuous fermentation of the thought of intelligence, artificial intelligence has extended its tentacles into the field of artistic creation and has begun to try intelligent creation. Painting creation based on artificial intelligence is called “intelligent painting.” For oil paintings, the computational language is a relatively complicated description. How to correctly identify the computational language of oil paintings is essential for establishing a large oil painting database. This paper constructs a meaningful learning similarity measure and multiclassification model based on the CCNN model to realize the classification of oil painting language. A cropped CNN model is used to extract language features, and on this basis, oil painting works are cross-compared and multiclassified. This method realizes the classification of oil painting language and the corresponding painter and achieves superior accuracy. This paper constructs a data classification method based on small samples, measures similarity through cross-comparison, and provides a measuring approach for classifying the language of oil paintings. The CCNN model proposed combines the best classification results of oil painting language, which improves the accuracy of oil painting language classification. Moreover, it further enriches the methods of oil painting language classification and image recognition under computational intelligence.

## 1. Introduction

As a unique symbol of artistic expression, oil painting language conveys the artist's understanding of the objective world and life and his feelings about life. It is a silent language, a bridge between the work and the audience's appreciation, and the basic expression of oil painting [1]. Language category is an important aspect of the meaning of an oil painting. For example, a portrait of Pierre-Auguste Renoir (1841–1919) painted in heavy ink and color, or a beautiful landscape painting by Claude Monet, shows the meaning of the work and the painter's thoughts. Famous artists often have their own unique language of their work, and understanding the language of art is crucial to artistic cognition. In order to better understand work of art, researchers put considerable effort into categorizing the language of work of art.

In computer vision research field, significant progress has been made in the classification of scenes in images and videos due to the improvement of machine learning algorithms and the improvement of image processing technology. Researchers have begun to try to automatically classify the language of work. Research on the use of machine learning methods to classify the language of work involves calligraphy, music, painting, etc. Among them, oil painting image classification can be regarded as two-dimensional signal classification.

Artistic language recognition in oil painting has their own characteristics in image features such as color, texture, and stroke thickness due to different painters [2]. They are hard to detect using traditional algorithms and objective characteristics. This research seeks to establish a CCNN-based oil painting language similarity measurement and classification method. The research done for this study in the language classification of oil paintings will be a new attempt and possibility for image classification technology.

## 2. Literature Review

Different painting styles, tools, and materials are all important factors that affect painting recognition. The styles of painters in different eras are extremely different, which is closely related to the artistic movements carried out in different periods [3]. For example, Gauguin, the representative of Post-Impressionism, pays more attention to color harmony rather than contrast when painting. Van Gogh, who is also a postimpressionist, emphasizes the use of colors to highlight themes, and his paintings are always a carnival of colors and brushstrokes.

Painting language classification is a field that people take an interest in. It can apply to painted identification, style classification, and painting database search. The task of painter classification includes classifying a painting by its respective painter. The style classification is a matter of classifying artists based on art schools such as Cubism, Baroque, and Impressionism. Both tasks are expected to be extremely challenging, because even the same painter's work has extremely different styles. Image processing techniques are a quantitative analysis tool that can perform scientific statistics and evaluation of works of art [4, 5]. Stork's comments on this topic use low-level functions that encode colors, shadows, textures, and edges [6]. Lombardi proposed that the style of painting is regarded as a personal label for the artist. Different controlled and uncontrolled teaching methods were utilized to study artists' classification tasks [7]. Lee and Cha proposed using color-based statistical calculations to extract global features and to classify painting styles by segmenting painting objects to extract local features based on composition [8]. On the basis of extracting features, using SOM to classify and visualize painting work provides a new way for artistic quantification.

Research on oil painting language classification has not made much progress in recent years. The main reason is that there is no unambiguous definition of artistic style. Numerous studies have transformed the recognition of oil painting language into the analysis of oil painting's color, texture, composition, and other characteristics and analyzed these specific characteristics in the classification model [9]. However, the results achieved are not good. The definition of language is subjective, and there is no definite quantitative indicator for the language distinction of oil paintings, so it is difficult to say in which aspects the differences between the two works are manifested [10].

In traditional oil painting language classification research, researchers extract oil painting features and then classify the features by modeling. Lombardi developed a system to study the problem of artistic analysis. Quantitative method was used to analyze the characteristics of oil paintings using the work of Van Gogh and painters of the same period as research samples, which are Van Gogh's unique brushstroke language. This paved the way for science [7]. Saleh et al. proposed a measurement method for figuring out the impact of different paintings to evaluate by learning how to optimize the primary functions of HOG [11]. Condorovici et al. studied the classification of Iranian paintings according to texture features. The characteristics of LBP, LPQ, and LCP oil paintings were extracted and input into KNN, SVM, and other models for classification. Recognition accuracy of painting language using SVM classifier is 96.24%, 96.42%, and 96.5% [12]. In the study of deep learning painting language classification, Bar et al. classified images according to the features obtained by the pretrained convolutional neural network for image classification tasks. A machine was designed to make aesthetically relevant semantic decisions. According to the existing consensus in the field of art history interpretation, predict painting language, genre and artist, and provide the best similarity measure [13]. In addition, 81449 images of 1119 artists were employed in the experiment, and the recognition accuracy reached 43%. In 2016, Tan et al. used neural network method to realize the automatic classification of oil painting language for the first time. They achieved an accuracy of 54.5% in the classification of more than 25 painting languages in Wiki-painting [14]. Lecoutre et al. suggested using deep residual nerve to solve the problem of discovering painting language, and the accuracy rate on the Wiki-paintings data set (for 25 different languages) reached 62% [15].

## 3. Methods

### 3.1. Convolutional Neural Networks (CNN)

In recent years, in the field of large-scale image classification, CNN has significant advantages, so it has attracted people's attention in the computer vision field. CNN is composed of a collection of multilayer neurons, which process the input image hierarchically [16]. Each layer can be seen as a collection of image filters, where each filter extracts specific characteristics from the input image.

CNN's research history goes back nearly 50 years, from the concept of prescription drugs in biological research in the 1960s to the first model based on prescription drug yards in the 1980s [9]. In 1998, Lecun of New York University proposed that the LeNet-5 model PW has made breakthrough progress in handwriting recognition, which has promoted the process of CNN research [17]. In recent years, CNN research in the field of pattern recognition has come to peak [18]. The above model resulted in the machine recognition error rate of ImageNet gallery approaching 3%, which is equivalent to the error rate of human eye recognition [17, 19, 20]. Although these models' structure is getting more and more complex, the bases of these models are almost the same.

CNN model is composed of three layers, that is, convolutional layer, a pooling layer, and a fully connected layer [21]. The convolutional layer is composed of a series of filter banks, which is of some size. And the depth of the filter is equal to the depth of the input data. The initial parameters can be set randomly, and parameters are updated in the training process.

Most of the current CNN structures will have a pooling layer embedded in it. Generally, a pooling layer is inserted into the fixed structure after the convolutional layer [19]. The pooling layer has a role of reducing the spatial size of the input data, so that it can reduce the possibility. The number of learning parameters greatly reduces the resource consumption of the computer and can also effectively control the overfitting of the training network [22].

Like a normal neural network, the neurons in the CNN are fully connected to input data of the former layer. The fully connected layer mainly maps the previously learned distribution features to the sample in the space; it plays a role of classification [23]. However, due to the parameter redundancy of the fully connected layer, some models (ResNet, GoogLeNet) have adopted other methods to replace the fully connected layer to avoid parameter redundancy and have achieved good results.

### 3.2. Information-Based Similarity (IBS)

The IBS method combines classification methods based on information and word statistics and is a novel method for effectively classifying symbol sequences based on word frequency and rank statistics [24]. In the research of natural language, it is found that each writer tends to use certain high-frequency words in the word citations in his writing, and the high-frequency words preferred by different writers differ from each other [25]. In 2003, Yang et al. applied this concept to the study of heart rate signals and, for the first time, proposed a method for similarity measurement of two complex sequences [26], which is the prototype of the IBS method. At present, IBS has been successfully applied in various fields, including Interbeat (RR) interval analysis, SARS coronavirus classification, and literary work research [24, 27].

The IBS method is to quantify the degree of difference between two symbol sequences. This degree of difference can be measured by the comparability of repeated patterns, and then the “distance” between the two symbol sequences is defined by the degree of difference. The steps for measuring the degree of difference between two symbol sequences are as follows:

(1) A sliding window of length *m* is applied to one of the symbol sequences Ψ_1_, and the sliding window divides the symbol sequence into a “word” of length *m*;
(2) Calculate the number of occurrences of each “word” and sort these words according to the number of occurrences. The more frequent the occurrence, the higher the ranking;
(3) Step (1) and step (2) are applied to another symbol sequence Ψ_2_ to obtain the sequence of the symbol sequence “words.” It can be seen that different symbol sequences correspond to different “words” sequence;
(4) According to the distribution of the “words” of the two symbol sequences Ψ_1_, Ψ_2_, the weight distance formula (1) is used to calculate the difference degree Dm of the two symbol sequences.
  (1) $$\begin{array}{c} {\text{D}}_{m}\left(\Psi_{1},\Psi_{2}\right)=\frac{1}{L}\sum_{k=1}^{L}\left|R_{1}\left(s_{k}\right)-R_{2}\left(s_{k}\right)\right|F\left(s_{k}\right). \end{array}$$

In formula (1), *s*_*k*_ is a “word” of length *m*; *R*_*i*_(*s*_*k*_) is the rank of a specific word *s*_*k*_ in the symbol sequence Ψ_*i*_; *L* represents the type of a word of length *m*, which is 2^m^ for a two-dimensional symbol sequence; *F*(*s*_*k*_) represents the weighting factor of the word *s*_*k*_.

When Yang et al. first proposed the similarity measurement theory [26], the weighting factor used is as shown in formula (2), and the weighting factor *F*_0_(*s*_*k*_) represents the proportion of the occurrence probability of the word *s*_*k*_ in the two symbol sequences Ψ_1_, Ψ_2_.

(2) $$\begin{array}{c} F_{0}\left(s_{k}\right)=\frac{p_{1}\left(s_{k}\right)p_{2}\left(s_{k}\right)}{z_{0}\sum_{k=1}^{L}p_{1}\left(s_{k}\right)p_{2}\left(s_{k}\right)}. \end{array}$$

In formula (2), *p*_*i*_(*s*_*k*_) refers the probability of the occurrence of a specific word *s*_*k*_ in the symbol sequence Ψ_*i*_. *Z*_0_ is a normalization factor, which is used to ensure that the difference degree D_*m*_ takes a value in the interval [0,1]. The definition of *Z*_0_ is as follows:

(3) $$\begin{array}{c} Z_{0}=2^{m}-1. \end{array}$$

In order to apply this difference index to the classification of more symbol sequences, Goldberger and Peng [24] proposed to use the Shannon entropy formula to redefine the weighting factor *F*(*s*_*k*_), as in formula (4), to obtain the difference, a new definition of the degree index.

(4) $$\begin{array}{c} F\left(s_{k}\right)=\frac{\left[-p_{1}\left(s_{k}\right)\log\;p_{1}\left(s_{k}\right)-p_{2}\left(s_{k}\right)\log\;p_{2}\left(s_{k}\right)\right]}{Z}. \end{array}$$

The normalization factor *Z* is defined as

(5) $$\begin{array}{c} Z=\sum_{k=1}^{L}\left[-p_{1}\left(s_{k}\right)\log\;p_{1}\left(s_{k}\right)-p_{2}\left(s_{k}\right)\log\;p_{2}\left(s_{k}\right)\right]. \end{array}$$

The steps for calculating the degree of difference between two sequences using the IBS method are as above. According to the definition of the degree of difference Dm, the more similar the two symbol sequences are in the order of words, the more similar the two sequences are. We can also visually reflect the “distance” between two symbol sequences by drawing a scatter diagram. If the words in the two sequences are more similar, the scatters are more concentrated near the diagonal. In some cases, the average deviation of the distance between these scattered points and the diagonal can be used to represent the “distance” between two sequences. The farther this “distance” is, the lower the similarity between the two sequences is.

Combining the distance measurement method of the IBS model and CNN, this research designs a CCNN model to measure and classify oil painting language.

### 3.3. Principles and Steps of CCNN

CCNN is built on CNN signal extraction and IBS distance measurement. The goal of CCNN is to determine the similarity of different images, so as to perform multi-image classification. The input data of CCNN are specific [28]. The algorithm uses deep-rolling neural network to extract features of images and generate a set of cross-probability maps through cross-comparison of images. In addition, CCNN defines a ModLBS distance to represent the cross similarity between probability map vectors based on IBS. When the distance of ModLBS is 1, it indicates that the reorganized image languages are different. A value of 0 means that the languages are similar and come from the same painter.

**Figure 1:**
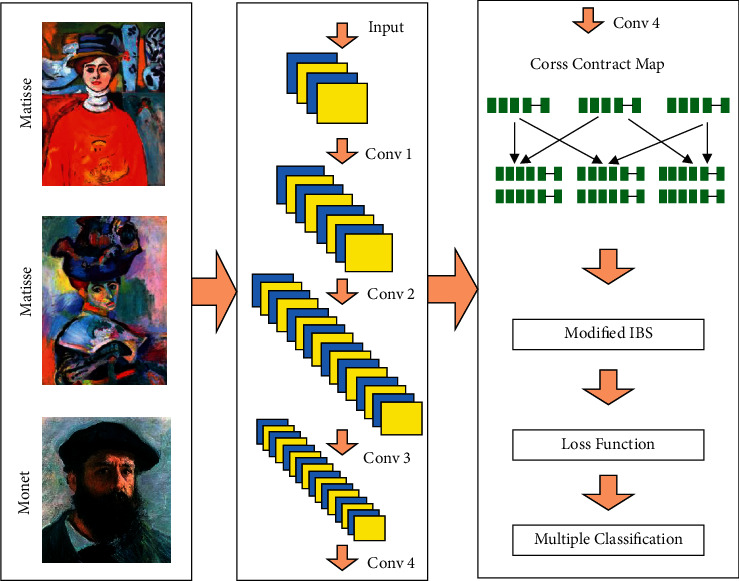
Schematic diagram of CCNN structure.

CCNN extracts features by comparing the implicit information generated by the input image and obtains a set of cross-probability maps. The purpose of distance measurement is to determine the similarity between images. The two are related to each other, and together, they form a complete network [29]. As shown in Figure 1, the network uses cnN-tuned image extraction mode and ModLBS to measure the similarities between the two images.

#### 3.3.1. Feature Extraction

First, introduce the part of the convolutional neural network used for feature extraction. The input image will be preprocessed before convolution and then pass through different layers of convolutional neural networks. The role of the convolutional layer is to do inner product operations, using multiple the convolution kernel operates on the pixels of a block. The convolution kernel is 3 × 3, the step size is 1, and the padding is 1. The convolution output is activated by ReLU and then pooled. The kernel size of the pooling layer is 2 × 2, so after the pooling layer, the input feature map will be cropped to 1/4 of the original, and the output of the pooling layer is the compressed feature map (feature map).  Table 1 shows the convolutional layer parameter changes in the convolution process, the ReLU layer is omitted in the table. It should be pointed out that, during the training and testing process, the input is a single image rather than a pair. Therefore, this paper can load the trained weight to initialize the CCNN, and no need to perform parameters. Here, this paper uses parameters trained on the ImageNet dataset of VGG19 to initialize the model.

#### 3.3.2. Cross-Comparison of Feature Images

After the convolutional neural network extracts the features, this paper will then perform cross-comparison and similarity measurement of the extracted features. First, it assumes that different convolution kernels correspond to different image features, and then the above output based on CNN feature extraction is a set of features.

The number of image features corresponds to that of convolution kernels. This paper first defines a threshold to vectorize the output feature images. The /(*v*) function is used to convert the output of the convolutional neural network into a probability distribution map, so that this paper can calculate the frequency of the feature represented by each convolution kernel. Here, *Y* is the *i*-th image of convolution filter in the *j*-th pixel value. *N* refers the number of image inputs. *D* represents the number of convolution kernels; *K* represents the output value of the last convolution layer.

(6) $$\begin{array}{c} {\overset{⟶}{p}}_{n}={\left[p_{1},p_{2},\ldots ,p_{i},\ldots ,p_{D}\right]|}_{n},n\in \left[1,N\right],\\ p_{i}=f\left(v_{i}\right)=\frac{\sum_{j}\text{softsign}\left(\max\left(v_{ij},0\right)\right)}{\sum_{i}\sum_{j}\text{softsign}\left(\max\left(v_{ij},0\right)\right)},\;\begin{array}{c} i\in \left[1,D\right],\\ j\in \left[1,K\right]. \end{array} \end{array}$$

**Figure 2:**
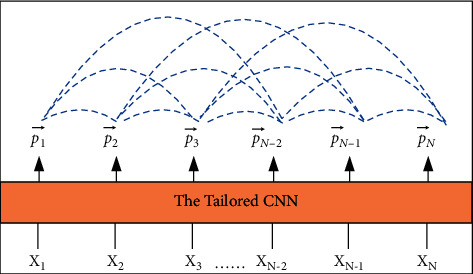
Schematic diagram of cross-contrast probability generation.

This paper constructs feature vectors in the output feature images and then combines them into multiple sets of sample pairs, so that the output of the corresponding convolutional neural network can be converted into many feature vectors with dimensions of (2, D).  Figure 2 fully shows the cross-comparison structure. Among them, *X*_*i*_ is the *i*-th frame of a group of images; *p*_*i*_ is the probability. Combining the probability mapping pair, the pair label is set to 0 if both are in the same category; if they do not belong to the same category, the pair label is set to 1.

Therefore, *N* (*N* ≥ 2) images can generate *C*_*N*_^2^=*N* × (*N* − 1)/2 sample pairs.

After constructing the corresponding feature vector, the language classification problem is transformed into how to measure and classify this group of feature vectors. In this way, an image classification problem is converted to a similarity measurement problem based on feature vectors. The CCNN model proposed in this paper focuses on the similarity of oil painting language, rather than the difference in exact image pixels. Therefore, when doing similarity measurement, in this model, value-sensitive Euclidean distance/Manhattan distance is not included. An improved IBS method is proposed to calculate the distance obtained by weighting information entropy. CCNN uses traditional IBS. Redefine it as NorlBS, and set a RevlBS value randomly.

#### 3.3.3. Similarity Measurement

Based on the idea of IBS, this paper first defines a NorlBS distance. 5 cores are the sum of the entropy of the probability distribution of the two images. NorlBS tends to 0 if they are similar; when they are completely different, it is not positive for the upper limit. Analogous to the principle of IBS, if the two images correspond to the same filter, and the output feature maps are ranked similarly. The scattered points are located close to both sides of the diagonal. The greater the distance, the smaller the similarity. The improved IBS method is based on the following assumption: if a feature is randomly shuffled, and the corresponding ranking changes significantly, then the fluctuation of the feature mapping may not be random and may contain relevant structural information. So, it is necessary to set a cross probability. Assuming that, after random destruction, distribution is still discrete, then the distance between the points after scrambling is defined as RevIBS.

(7) $$\begin{array}{c} \text{NorIBS}=\frac{\sum_{i}\left|R_{xi}-R_{yi}\right|SE_{i}}{D{\sum_{i}SE}_{i}},\\ SE_{i}=E_{x}\left(p_{i}\right)+E_{y}\left(p_{i}\right),\\ E\left(p\right)=-p\;\log\;p,\\ \text{RevIBS}=\text{NorIBS}\left(\text{random}\left(\overset{⟶}{p_{x}}\right)\right). \end{array}$$

For the two to work in different languages, that is, when the similarity category label is 1, the distribution before and after scrambling does not change much, so the corresponding ResIBS and NorlBS have not changed much. This paper can think that the difference between the two has a tendency to 0. And for two paintings of the same painter, the RevIBS value after scrambling is greater than NorlBS, and the difference between the two is greater than 0. Therefore, this paper defines a distance ModIBS as follows (8), where *P* is a coefficient, which is in 2∼4. ModIBS is in 0∼1. For ModIBS, when it tends to 0, the languages of the two works are similar. The value tending to 1 means that the languages of the two paintings are extremely different.

(8) $$\begin{array}{c} \text{ModIBS}=e^{-{\left(\text{RevlBS}-\text{NorIBS}/\text{NorIBS}\right)}^{\beta }}. \end{array}$$

Finally, this paper defines a loss function and establishes the optimization goal of the model in this paper to complete the construction of the model. The loss function is as follows, where *y* is the category similarity label, and *y* = 0, indicating that the two oil paintings are the same painter, and vice versa, and *y* = *l* means different.

(9) $$\begin{array}{c} L=\sum -\left[y\;\log\left(\text{ModIBS}\right)-\left(1-y\right)\log\left(1-\text{ModIBS}\right)\right]. \end{array}$$

### 3.4. Data Set

To train the model, the Wiki-paintings dataset is used. It is a large-scale image dataset collected by Karayev et al. of WikiArt [30]. The Wiki mapping dataset contains more than 85,000 images, which are divided into 25 languages/genres, such as Abstract Art, Abstract Expressionism, Baroque, Color Field Painting, Cubism, Early Renaissance, and Expressionism. The styles of oil painting pictures from the Wiki-paintings dataset include Academicism, Baroque, Expressionism, High Renaissance, Low Renaissance, Impressionism, neoclassicism, Primitivism, Realism, and Rococo.

Some paintings can have similar content, but different languages. This paper selects 20 artists in different languages to form the “Selected-Wiki-Painting” data set of this paper. The distribution of genres, artists, and the number of works is shown in Table 2.

**Figure 3:**
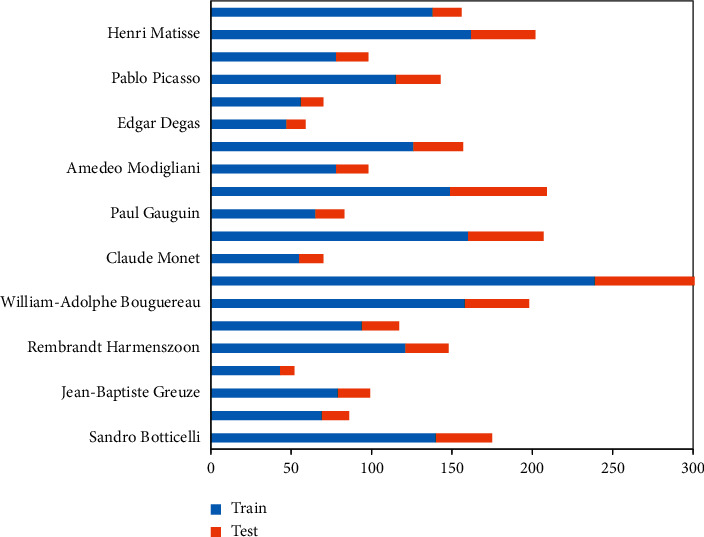
Distribution of oil painting data sets.

Each artist contains more than 50 pictures. The specified train/test ratio is about 4 : 1. As can be seen from Figure 3, Renior has the largest number of paintings in the data set, and the number of images drawn by Vermeer is drawn by these 20 artists. At least, in order to ensure that the model is based on the classification of the oil painting language, the selected images are all portraits, which guarantees the general similarity of the content.

## 4. Result and Discussion

### 4.1. Data Preprocessing

Data preprocessing has a greater impact on the quality of the model, and different data sets and different preprocessing methods should be used. Common image preprocessing methods in deep learning include the following three:

Zero mean: subtract the average value of each dimension of the original data of each dimension, and replace the original data with the result.

(10) $$\begin{array}{c} x_{\text{new}}=x-\mu . \end{array}$$

Min-max standardization: it is a linear transformation of the original data in [0,1] by mapping, and its mathematical expression is as follows:

(11) $$\begin{array}{c} x_{\text{new }}=\frac{x-x_{\min}}{x_{\max}-x_{\min}}. \end{array}$$

Z-Score standardization: the data after Z-Score standardization conforms to the standard normal distribution.

(12) $$\begin{array}{c} x_{\text{new}}=\frac{\bar{x}-\mu }{\delta }. \end{array}$$

In the CCNN-based oil painting language and artist classification process, data preprocessing mainly includes the following steps:

- Crop the original image to a uniform size (224, 224, 3);
- Perform min-max standardization on the cropped image, and normalize the pixel value of the image to [0,255];
- Zero-average processing is performed, and each pixel of the three channels of each image is subtracted from its corresponding RGB three-channel pixel average.

Zero-average processing can remove the average brightness value of the image and pay more attention to the image language or content, and at the same time, data distribution with 0 center is also constructed, so that the gradient descent algorithm can effectively converge.

### 4.2. Model Training

This section conducts experiments on the data set selected, that is, Wiki-painting. VGG19 model pretrained on ImageNet is used to initialize the parameters. In addition, some related strategies are used, such as early stopping, learning rate, and index decay.

**Figure 4:**
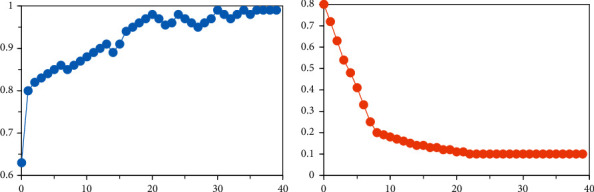
Accuracy and loss changes during the training. (a) The classification accuracy change. (b) The loss change.

Implementation of the CCNN model is based on the TensorFlow framework. Model training includes image preprocessing, model initialization and training, parameter tuning, and other steps. Model training has two parts: forward propagation and backpropagation. In the backpropagation process, CCNN uses tf.train.AdamOptimizer provided by TensorFlow to update its parameters. Taking this into account, CCNN iterates the model parameters. The network gradually approaches the optimal solution and finally achieves the optimal goal. Here, the Adam method is a method founded on gradient descent. The tf.train.AdamOptimizer provided by TensorFlow can improve traditional gradient descent by using momentum, promote dynamic adjustment of hyperparameters, and accelerate model convergence. In addition, the CCNN model uses L2 regularization to prevent overfitting, and through loss, the sum of the squares of the parameter *w* is added to suppress the value of *w* and make the loss curve smoother, thereby reducing overfitting. The specific implementation of L2 in TensorFlow is tf.add_n(tf.get_collection(‘losses')),weight). Extract all loss and use total_loss to train. In Figure 4, the accuracy and loss of the training set data are changing during the model training process (lr = 4.261487e-05, batch_size = 21).

**Figure 5:**
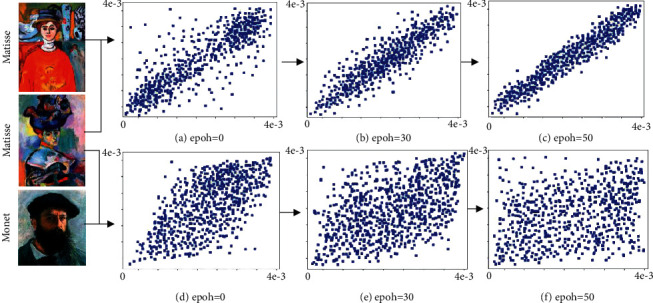
The probability distributions of different images.

Figure 5 shows the probability graph of similarity between two paintings by Matisse and a painting by Matisse and a painting by Monet. It is found that, in the initial stage, the probability distribution of the two paintings of different authors in the picture tends to be scattered. As the number of training increases, the degree of scatter increases. On the contrary, for the two paintings of the same artist, as the training goes, it will be better distributed on both sides of the central axis. Observe the loss curve. As the training increases, the average loss is steadily decreasing, which indicates that the model training is converging in the expected direction.

### 4.3. Model Test

**Figure 6:**
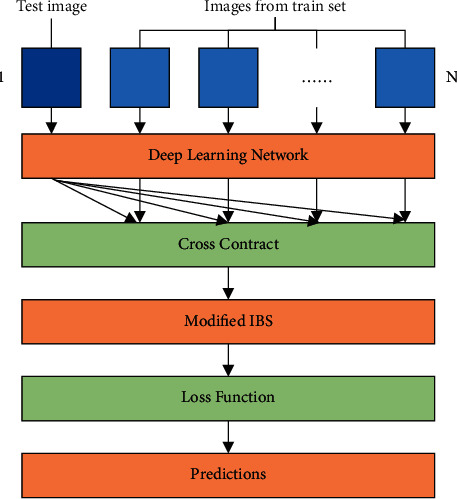
CCNN test method.

As can be seen from Figure 6, the test process of CCNN oil painting language classification is as follows. One test image and *n* training set images are combined into a set of inputs, and input into the trained model. 1 test image and 1 one training set image can get 1 ModIBS value, and *n* training set images can get *n* ModIBS values.

Three accuracy indicators are included in the model. Similarity accuracy (SA) is defined as the predictive accuracy of all pairs used to determine whether two images are similar, that is, two classifications. LVA is to select the category containing more label0 as the final classification result and calculate the accuracy rate. DVA is to select the category with the smallest average IBS modification value as the final classification. LVA and DVA aim to evaluate the classification accuracy of multiple classifications.

### 4.4. Classification Result

In the classification task, the goal is to find the similarity between two input images and realize its multiclassification. The model is applied to the identification and language classification of 20 painters. Results are presented in Table 3. Among them, in the classification of artists, the accuracy rate of similarity measurement is 97.95%. The DVA and LVA were 85.75% and 80.26%, respectively. The results of classification and recognition show that the model can effectively classify the two images. In other words, CCNN can estimate the structure from the contrasted images, copying the process of the human brain in measuring these images. In the classification of art genres, the accuracy rate of its 13 genres multiclassification is the highest 52.77%.

## 5. Conclusion

How to correctly identify the artistic language of oil paintings is essential for establishing a large oil painting database. The classification of artistic language cannot rely on any concrete characteristics, especially for those artistic works that are not representative. In response to the above problems, this paper proposes the CCNN model. The model performs well in the multiclassification task of oil painting language. This paper proposes a CCNN multiclassification method, which can measure the similarity and multiclassification of the language of oil painting work and the corresponding artists. Oil painting data set was constructed using the WikiArt library. The data set includes more than 2,000 works by 13 language styles and 20 artists. CCNN achieved an accuracy of 85.75% on the task of artistic classification. The CCNN model proposed dividing the 13 languages of the oil painting data set finds the best combination of the classification results and improves the accuracy of oil painting language classification. Although the CCNN model can be well applied to multiclassification tasks of small samples with obvious language features, its depth should be further expanded, such as extending ResNet for feature extraction.

## Figures and Tables

**Table 1:** VGGNet network structure of each level.

Input	Layer name	Array size
C-3-64	C 1–1	224 × 224 × 3
C-64-64	C 1–2	224 × 224 × 64
Maxpool	Pool1	112 × 112 × 64
C-64-128	C 2–1	112 × 112 × 128
C-128-128	C 2–2	112 × 112 × 128
Maxpool	Pool2	56 × 56 × 128
C-128-256	C 3–1	56 × 56 × 256
C-256-256	C 3–2	56 × 56 × 256
C-256-256	C 3–3	56 × 56 × 256
C-256-256	C 3–4	56 × 56 × 256
Maxpool	Pool3	28 × 28 × 256
C-256-512	Conv 4–1	28 × 28 × 512

**Table 2:** The number of different genres, painters and works.

Art genres	Painters	Train	Test	Total
Early renaissance	Sandro Botticelli	140	35	175
High renaissance	Leonardo da Vinci	69	17	86
Rococo	Jean-Baptiste Greuze	79	20	99
Baroque	Johannes Vermeer	43	9	52
	Rembrandt Harmenszoon	121	27	150
Neoclassicism	Auguste Dominique Ingres	94	23	117
	William-Adolphe Bouguereau	158	40	198
Impressionism	Pierre-Auguste Renoir	239	66	305
	Claude Monet	55	15	70
Post-impressionism	Vincent Willem van Gogh	160	47	207
	Paul Gauguin	65	18	83
Expressionism	Egon Schiele	149	60	209
	Amedeo Modigliani	78	20	98
	John Singer Sargent	126	31	157
Realism	Edgar Degas	47	12	59
	Jean-Francois Millet	56	14	70
Surrealism	Pablo Picasso	115	28	143
Symbolism	Odilon Redon	78	20	98
Fauvism	Henri Matisse	162	40	202
Else	Nicolai Fechin	138	18	156
Total	20	2172	560	2732

**Table 3:** Oil painting language classification results based on CCNN.

Classes	Artists/Style	SA (%)	DVA (%)	LVA (%)
13	Styles	86.96	52.77	47.76
20	Selected-wiki paintings	97.95	85.75	80.26

## Data Availability

The dataset can be accessed upon request.
